# Acute dose-dependent effects of mescaline in a double-blind placebo-controlled study in healthy subjects

**DOI:** 10.1038/s41398-024-03116-2

**Published:** 2024-09-30

**Authors:** Aaron Klaiber, Yasmin Schmid, Anna M. Becker, Isabelle Straumann, Livio Erne, Alen Jelusic, Jan Thomann, Dino Luethi, Matthias E. Liechti

**Affiliations:** 1 Clinical Pharmacology and Toxicology, Department of Biomedicine and Department of Clinical Research, University Hospital Basel, Basel, Switzerland; 2 Department of Pharmaceutical Sciences, University of Basel, Basel, Switzerland

**Keywords:** Pharmacology, Human behaviour

## Abstract

Classic psychedelics have regained interest in research and therapy. Despite the long tradition of the human use of mescaline, modern data on its dose-dependent acute effects and pharmacokinetics are lacking. Additionally, its mechanism of action has not been investigated in humans. We used a randomized, double-blind, placebo-controlled, crossover design in 16 healthy subjects (8 women) who received placebo, mescaline (100, 200, 400, and 800 mg), and 800 mg mescaline together with the serotonin 5-hydroxytryptamine-2A (5-HT_2A_) receptor antagonist ketanserin (40 mg) to assess subjective effects, autonomic effects, adverse effects, and pharmacokinetics up to 30 h after drug administration. Mescaline at doses >100 mg induced dose-dependent acute subjective effects. Mescaline increased systolic and diastolic blood pressure at doses >100 mg, with no difference between doses of 200-800 mg. Heart rate increased dose-dependently. Pharmacokinetics of mescaline were dose-proportional. Maximal concentrations were reached after approximately 2 h, and the plasma elimination half-life was approximately 3.5 h. The average duration of subjective effects increased from 6.4 to 14 h with increasing doses of 100-800 mg mescaline. Nausea and emesis were frequent adverse effects at the 800 mg dose. Co-administration of ketanserin attenuated and shortened acute effects of 800 mg mescaline to become comparable to the 100 and 200 mg doses. There were no ceiling effects of the subjective response within the investigated dose range, but tolerability was lower at the highest doses. These results may assist with dose finding for future research and suggest that acute effects of mescaline are primarily mediated by 5-HT_2A_ receptors.

## Introduction

Mescaline (3,4,5-trimethoxyphenethylamine) is a serotonergic psychedelic with a long history of ethnomedical use [1, 2]. Other classic psychedelics include lysergic acid diethylamide (LSD), psilocybin, and *N,N*-dimethyltryptamine (DMT), which have regained interest in psychiatric research. LSD and psilocybin are particularly being investigated as treatments for depression and anxiety-related disorders [3–7]. Mescaline is found as an alkaloid in different species of cacti (e.g., peyote or San Pedro) in North and South America. The radiocarbon dating and alkaloid analysis of archeological specimens of peyote buttons revealed that mescaline was used for more than 5000 years by native North Americans [8]. Subjective effects of mescaline were scientifically investigated and described starting at the end of the 19^th^ century and throughout the 20th century [9–13]. In contrast to other classic psychedelics, there has been little contemporary clinical research on mescaline. A recent first study determined the dose-equivalence of mescaline, LSD, and psilocybin and found no differences in the overall quality of subjective effects between these three classic psychedelics [14]. However, no modern data are available on the acute psychedelic effects, tolerability, and pharmacokinetics of different doses of mescaline in humans. Therefore, we investigated acute subjective, autonomic, and adverse effects of mescaline across a range of doses and within-subjects in healthy participants. The selected range covers doses from very low to very high, based on previous studies with mescaline [2, 12, 14–16]. Additionally, we fully characterized the pharmacokinetics of different doses of mescaline. The psychedelic effects of LSD, psilocybin, and DMT have been shown to be mediated primarily by serotonin 5-hydroxytryptamine-2A (5-HT_2A_) receptors in humans. Specifically, the 5-HT_2A_ receptor antagonist ketanserin prevented [17–20] or reversed [21] most acute subjective effects in healthy subjects. No studies have yet similarly explored the mechanism of action of mescaline in humans. Hence, we investigated the role of 5-HT_2A_ receptors in acute effects of mescaline by administering a high dose of 800 mg mescaline with either the 5-HT_2A_ receptor antagonist ketanserin or placebo. The present study is the first contemporary full characterization of acute effects of different doses of mescaline that describes the role of 5-HT_2A_ receptors in mescaline-induced altered states in healthy volunteers. We hypothesized that effects of mescaline would be dose-dependent and blocked by ketanserin.

## Methods and materials

### Study design

The study used a double-blind, placebo-controlled, cross-over design with six experimental test sessions to investigate responses to (*i*) placebo, (*ii*) 100 mg mescaline, (*iii*) 200 mg mescaline, (*iv*) 400 mg mescaline, (*v*) 800 mg mescaline, and (*vi*) 800 mg mescaline plus 40 mg ketanserin that were administered in a counter-balanced order. The washout periods between sessions were at least 14 days. The study was conducted in accordance with the Declaration of Helsinki and International Conference on Harmonization Guidelines in Good Clinical Practice and approved by the Ethics Committee of Northwest Switzerland (EKNZ) and Swiss Federal Office for Public Health. The study was registered at ClinicalTrials.gov (NCT04849013).

### Participants

Sixteen healthy subjects (8 men and 8 women; mean age ± SD: 33 ± 10 years; range: 25-55 years) were recruited by word of mouth. All subjects provided written informed consent and were paid for their participation. Exclusion criteria were age <25 years or >65 years, pregnancy (urine pregnancy tests were performed at screening and before each test session), personal or first-degree relative psychotic disorders, current or history of major psychiatric disorders (assessed by the Semi-structured Clinical Interview for *Diagnostic and Statistical Manual of Mental Disorders*, 4th edition, Axis I disorders), the use of medications that may interfere with the study drugs (e.g., antidepressants, antipsychotics, and sedatives), chronic or acute physical illness (e.g., abnormal physical exam, electrocardiogram, or hematological and chemical blood analyses), tobacco smoking (>10 cigarettes/day), lifetime prevalence of hallucinogenic drug use >20 times (except for Δ^9^-tetrahydrocannabinol), illicit drug use within the last 2 months, and illicit drug use during the study period (determined by randomly performed urine drug tests). The participants were asked to consume no more than 20 standard alcoholic drinks/week and have no more than one drink on the day before the test sessions.

Thirteen participants had previously used a psychedelic (3-15 times), including LSD (11 participants), psilocybin (10 participants), DMT (three participants), and mescaline (three participants). Nine participants had used 3,4-methylenedioxymethamphetamine (MDMA, 2-10 times). Ten participants had used a stimulant (1-15 times), including methylphenidate (two participants), amphetamine (three participants), and cocaine (nine participants). Two participants had used 4-bromo-2,5-dimethoxyphenethylamine (2C-B; 1-2 times). Two participants had used ketamine (1-5 times). Six participants had used nitrous oxide (1-5 times). One participant had never used any illicit drugs with the exception of cannabis.

### Study drugs

Mescaline hydrochloride (ReseaChem GmbH, Burgdorf, Switzerland) was administered in capsules that were produced according to Good Manufacturing Practice in dosing units that contained 100 mg mescaline hydrochloride. Ketanserin was obtained as the marketed drug Ketensin (20 mg, Janssen-Cilag, Leiden, NL) and encapsulated with opaque capsules to ensure blinding. Placebo consisted of identical opaque capsules that were filled with mannitol. See Supplementary Methods online for details. At the end of each session and at the end of the study, the participants were asked to retrospectively guess their treatment assignment.

### Study procedures

The study included a screening visit, six 31-h test sessions (each separated by at least 14 days), and an end-of-study visit. The sessions were conducted in a calm hospital room. Only one research subject and one investigator were present during each test session. The test sessions began at 8:00 AM. A urine sample was taken to verify abstinence from drugs of abuse, and a urine pregnancy test was performed in women with child-bearing potential. The subjects then underwent baseline measurements. Ketanserin (40 mg) or placebo and mescaline or placebo were administered at 9:00 AM. The outcome measures were repeatedly assessed for 30 h after drug administration. A standardized lunch and dinner were served. The subjects were never alone during the first 16 h after drug administration, and the investigator was in a room next to the subject for up to 30 h. The subjects were sent home the next day at 3:15 PM.

### Subjective drug effects

Subjective effects were assessed repeatedly using visual analog scales (VASs) [22, 23] and the Adjective Mood Rating Scale (AMRS) [24]. The 5 Dimensions of Altered States of Consciousness (5D-ASC) scale [25, 26] was administered at the end of the session at 30 h after drug administration to retrospectively rate peak drug effects. Mystical experiences were similarly assessed 30 h after drug administration using the States of Consciousness Questionnaire (SCQ) [27, 28] that includes the 43-item Mystical Experience Questionnaire (MEQ43) [27], the 30-item MEQ 30 [29], and subscales for “aesthetic experience” and negative “nadir” effects. Subjective effects ratings are described in detail in the Supplementary Methods online.

### Autonomic and adverse effects

Blood pressure, heart rate, tympanic body temperature, and pupil size were repeatedly measured [30]. Adverse effects were assessed using the List of Complaints 1 h before and 14 and 30 h after drug administration.

### Plasma mescaline and ketanserin concentrations

Blood was collected into lithium heparin tubes at 0, 0.5, 1, 1.5, 2, 3, 4, 5, 6, 8, 9, 10, 11, 12, 14, 16, 20, 24, and 30 h after drug administration. The blood samples were immediately centrifuged, and plasma was subsequently stored at -80 °C until analysis. Plasma concentrations of mescaline and ketanserin were determined by high-performance liquid chromatography-tandem mass spectrometry [21, 31].

### Data analysis

Peak (E_max_ and/or E_min_), peak change from baseline (ΔE_max_ and/or ΔE_min_) values or area under the effect curve (AUEC) were determined for repeated measures. The values were then analyzed using repeated-measures analysis of variance (ANOVA), with drug dose as the within-subjects factor. Additional ANOVAs were performed with order as between-subjects factor. Tukey *post hoc* tests were performed based on significant main effects or interactions. For data analysis, the statistical analysis software R was used ([32]. The criterion for significance was *p* < 0.05. Pharmacokinetic parameters were estimated using non-compartmental models in Phoenix WinNonlin 6.4 (Certara, Princeton, NJ, USA) [33]. The time to onset, maximal effect, offset, effect duration, and AUEC were assessed in the any-drug-effect–time plots using a threshold of 10% of the maximum individual response. The AUEC was determined using linear trapezoidal with linear interpolation method in Phoenix WinNonlin 6.4 [33].

## Results

### Subjective drug effects

**Fig. 1:**
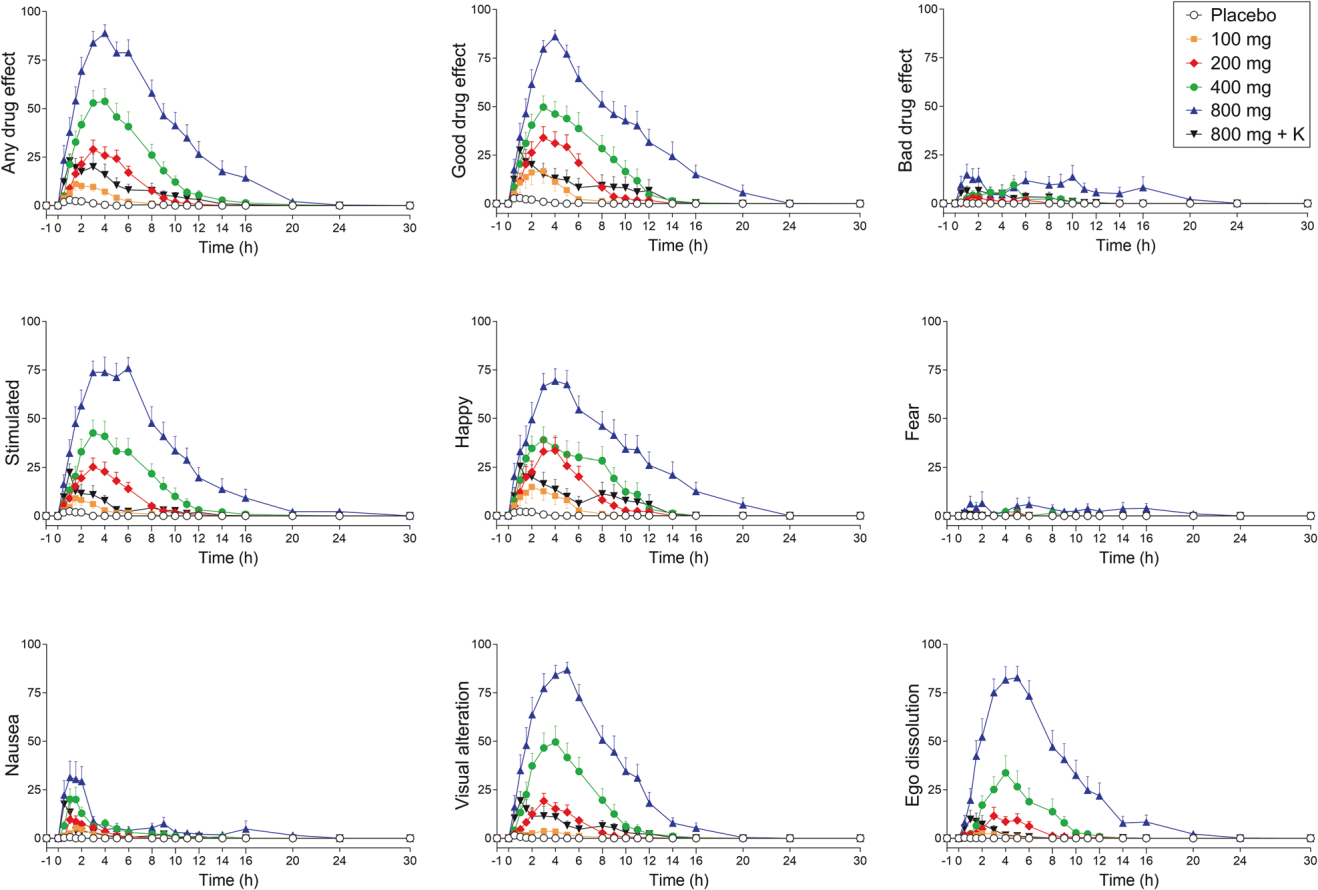
Acute subjective effects of different doses of mescaline over time. Mescaline elicited dose-dependent acute subjective effects compared with placebo. No ceiling effects were reached. The co-administration of ketanserin strongly reduced acute effects of mescaline (800 mg). Both mescaline (100–800 mg) and ketanserin (K; 40 mg) and their respective placebos were administered at *t* = 0 h. The data show the mean ± SEM of Visual Analog Scale ratings (0–100%) in 16 participants. Maximal responses and statistics are shown in Supplementary Table S1.

**Fig. 2:**
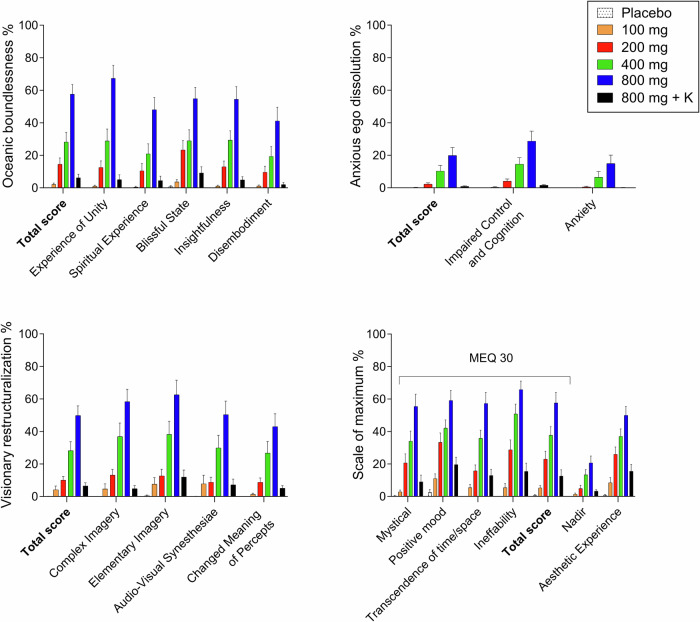
Acute alterations of consciousness on the 5 Dimensions of Altered States of Consciousness (5D-ASC) Scale and mystical-type experiences on the Mystical Experience Questionnaire (MEQ). Mescaline produced dose-dependent alterations of consciousness and mystical-type experiences compared with placebo, with significant changes at doses >100 mg. There was no ceiling effect. The co-administration of ketanserin (K) reduced acute alterations of consciousness and mystical-type experiences of mescaline (800 mg) to the level of 100–200 mg mescaline. The data show the mean ± SEM percentage of maximally possible scale scores in 16 participants. Statistics are shown in Supplementary Table S1.

Subjective effects over time on the VAS and AMRS are shown in Fig. 1 and Supplementary Figs. S1 and S2. The corresponding peak responses and statistics are presented in Supplementary Table S1. Alterations of mind on the 5D-ASC and mystical-type experiences on the MEQ 30 are presented in Fig. 2. Statistics are summarized in Supplementary Table S1.

On the VAS, mescaline produced dose-dependent subjective effects starting at the 200 mg dose. Significant bad drug effects occurred only at the 800 mg dose. Nausea increased at 400-800 mg compared with placebo (Fig. 1). Generally, higher doses produced proportionally greater subjective responses with no ceiling effect (Fig. 1 and Supplementary Table S1). The maximal effect, area under the effect-time curve, and effect duration of “any drug effect” consistently increased dose-dependently (Table 1). Ketanserin co-administration strongly reduced and shortened the subjective response to 800 mg mescaline to become similar to the effect of 100-200 mg mescaline (Table 1 and Fig. 1). Individual mescaline effect reductions by ketanserin along with plasma ketanserin levels in each participant are shown in Supplementary Fig. S4. No order effects were observed in the subjective response to mescaline, except for nausea.

**Table 1:** Characteristics of the acute subjective response to Mescaline.

Effect	Mescaline 100 mg	Mescaline 200 mg	Mescaline 400 mg	Mescaline 800 mg	Mescaline 800 mg + 40 mg Ketanserin
	*n* = 16	*n* = 16	*n* = 16	*n* = 16	*n* = 16
Time to onset (h)	0.5 ± 0.5^a^ (0.1–1.7)	0.6 ± 0.4 (0.1–1.2)	0.6 ± 0.5 (0.2–1.6)	0.5 ± 0.5 (0.1–1.8)	0.3 ± 0.3 (0.1–1.1)
Time to offset (h)	6.9 ± 2.2^a^ (3.9–11)	8.9 ± 1.6 (5.8–12)	11 ± 2.8 (8.1–19)	15 ± 3.9 (7.3–22)	7.9 ± 4.3 (1.5–17)
Time to maximal effect (h)	2.0 ± 1.3 (0.0–5.0)	3.0 ± 1.0 (1.0–5.0)	3.2 ± 1.3 (1.5–6.0)	3.4 ± 1.3 (0.5–6.0)	1.9 ± 1.4 (0.5–6.0)
Effect duration (h)	6.4 ± 2.0^a^ (3.0–10)	8.3 ± 1.7 (4.7–12)	11 ± 2.8 (7.9–19)	14 ± 3.7 (7.2–22)	7.7 ± 4.1 (1.4–17)
Maximal effect (%)	16 ± 13 (0–51)	33 ± 17 (10–74)	60 ± 26 (9–100)	92 ± 15 (50–100)	38 ± 23 (17–91)
AUEC	45 ± 38 (0–138)	154 ± 100 (44–429)	370 ± 216 (32–821)	817 ± 353 (318–1676)	147 ± 141 (24–519)

Parameters are for “any drug effect” as determined using the individual effect-time curves. The threshold to determine times to onset and offset was set to 10% of the individual maximal response. Values are mean ± SD (range); *AUEC* area under the effect curve; ^a^*n* = 14.

Mescaline dose-dependently increased alterations of mind and mystical-type experiences on the 5D-ASC and MEQ 30, respectively, starting at 200 mg compared with placebo (Fig. 2 and Supplementary Table S1). Anxiety on the 5D-ASC significantly increased only at the 800 mg dose. 5D-ASC and MEQ 30 ratings markedly and mostly significantly increased further from 400 to 800 mg, indicating no ceiling effect at the doses tested.

Ketanserin co-administration reduced 5D-ASC and MEQ 30 scores compared with 800 mg mescaline alone to levels that were reached with 100-200 mg mescaline (Fig. 2 and Supplementary Table S1).

### Autonomic and adverse effects

**Fig. 3:**
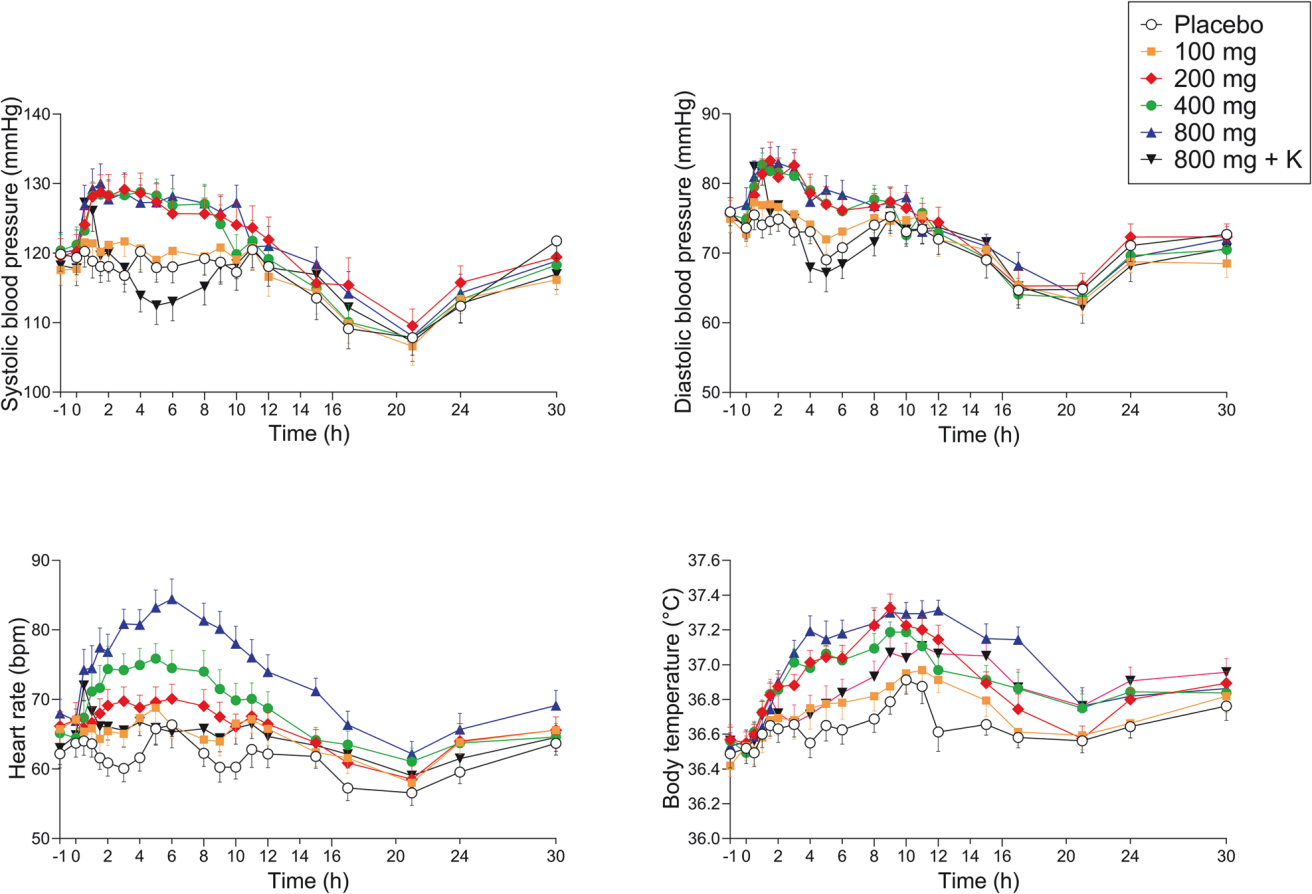
Acute autonomic effects of mescaline over time. Mescaline increased diastolic and systolic blood pressure at 200–800 mg compared with placebo and independent of dose. Mescaline dose-dependently elevated heart rate and body temperature at doses of 200–800 mg. Ketanserin decreased all autonomic effects of mescaline. Both mescaline (100–800 mg) and ketanserin (K; 40 mg) were administered at *t* = 0 h. The data show mean ± SEM values in 16 participants. Maximal responses and statistics are shown in Supplementary Table S1.

Autonomic effects over time are shown in Fig. 3. Maximal effects and statistics are shown in Supplementary Table S1. Adverse effects are listed in Supplementary Tables S1 and S3.

Mescaline increased systolic and diastolic blood pressure and body temperature relatively similarly at the 200-800 mg doses compared with placebo (Fig. 3). In contrast, mescaline increased heart rate more dose-dependently compared with placebo (Fig. 3). Co-administration of ketanserin reversed the mescaline-induced elevations of blood pressure and heart rate. The peak blood pressure responses to mescaline were not significantly reduced by ketanserin because the onset of the effect of ketanserin occurred after the mescaline-induced increase in blood pressure. The peak effect of mescaline (800 mg) on heart rate and body temperature was significantly reduced by ketanserin (Fig. 3 and Supplementary Table S1). Mescaline dose-dependently increased pupil size and reduced the light reflex compared with placebo. Ketanserin transiently reduced these mescaline-induced changes in pupillary function (Supplementary Fig. S3 and Supplementary Table S1). Mescaline dose-dependently induced acute (0–14 h) and subacute (14–30 h) adverse effects on the List of Complaints compared with placebo (Supplementary Tables S1 and S3). Frequent acute adverse effects included fatigue, headache, lack of concentration, and nausea (Supplementary Table S3). Mescaline caused emesis in two and seven subjects at the 400 and 800 mg doses, respectively. Ketanserin co-administration reduced the number of participants who experienced emesis to two. One subject reported having one flashback after the study day with 800 mg mescaline. [34]. Spontaneously reported adverse events during the study included headaches (3 subjects), stomachache (1 subject), dizziness (1 subject), and nosebleed (1 subject).

### Pharmacokinetics

**Fig. 4:**
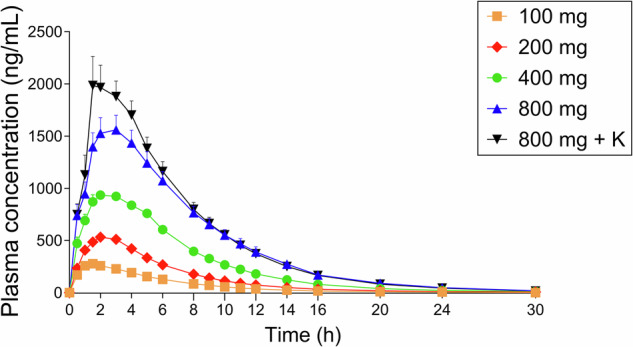
Plasma concentrations of different doses of mescaline. Plasma concentrations of mescaline increased in a dose-proportional manner. Plasma concentration of mescaline after the 800 mg dose was slightly lower than expected, likely due to emesis in 6 of 16 participants during onset of drug effects and potentially reduced availability of the orally administered amount of mescaline. When mescaline was administered together with ketanserin (K), nausea and vomiting were reduced (only one participant experienced emesis). Concentrations of mescaline were in the expected range (twice as high as with the 400 mg dose) when the 800 mg dose was administered with ketanserin. Mescaline and ketanserin were administered at *t* = 0 h. Values are mean ± SEM. Pharmacokinetic parameters are shown in Table 2.

Pharmacokinetic parameters of mescaline and its two main metabolites, 3,4,5-trimethoxyphenylacetic acid and *N*-acetyl mescaline, are shown in Table 2 and Fig. 4. Plasma mescaline concentrations increased proportionally with increasing mescaline doses (Fig. 4). However, the 400 and 800 mg doses of mescaline resulted in slightly lower concentrations than expected based on the 100 mg mescaline, 200 mg mescaline, and 800 mg mescaline + ketanserin conditions, likely because of more vomiting. The concentration of 800 mg mescaline was in the expected range when it was co-administered with ketanserin, which reduced vomiting.

**Table 2:** Pharmacokinetic parameters [geometric mean (95% CI), range] of parent substances and their metabolites.

	*C*_max_ (ng/mL)	*t*_max_ (h)	*t*_1/2_ (h)	AUC_30_ (ng h/mL)	AUC_∞_ (ng h/mL)	CL/F (L/h)	V_z_/F (L)
Mescaline 100 mg							
Mescaline	298 (268–332)	1.6 (1.3–2.0)	3.5 (3.2–3.8)	1767 (1660–1880)	1805 (1700–1916)	55 (52–59)	281 (254–312)
	205–420	1.0–4.0	2.6–4.4	1346–2038	1395–2063	49–72	205–380
TMPAA	277 (249–308)	1.6 (1.4–1.9)	4.0 (3.6–4.4)	1632 (1454–1830)	1676 (1496–1878)	60 (53–67)	346 (305–392)
	203–401	1.0–3.0	2.6–5.5	1068–2382	1109–2438	41–90	256–571
NAM	14 (8–25)	1.6 (1.4–1.8)	2.3 (2.1–2.6)	60 (32–113)	63 (34–117)	1588 (858–2939)	5372 (3147–9171)
	3.0–83	1.0–3.0	1.5–3.1	7.7–502	8.8–504	198–11,305	899–24,884
Mescaline 200 mg							
Mescaline	568 (522–619)	1.9 (1.5–2.3)	3.7 (3.4–4.0)	3588 (3342–3853)	3636 (3393–3898)	55 (51–59)	292 (266–321)
	444–696	1.0–4.0	2.6–5.1	2582–4737	2642–4775	42–76	224–404.9
TMPAA	530 (475–591)	1.9 (1.7–2.2)	4.1 (3.8–4.4)	3288 (2962–3650)	3344 (3021–3701)	60 (54–66)	351 (313–394)
	338–710	1.5–3.0	3.0–5.0	2253–4540	2331–4578	44–86	246–551
NAM	38 (22–66)	2.3 (2.0–2.7)	2.2 (1.9–2.5)	177 (95–332)	181 (98–336)	1105 (595–2049)	3496 (2051–5959)
	5.8–211	1.5–4.0	1.3–3.5	18–1351	20–1355	148–9995	587–19131
Mescaline 400 mg							
Mescaline	1034 (944–1132)	2.3 (1.8–2.9)	3.7 (3.4–3.9)	7435 (6791–8140)	7493 (6844–8204)	53 (49–58)	283 (258–311)
	692–1328	1.0–5.0	2.9–4.7	5443–10,491	5478–10,550	38–73	206–415
TMPAA	872 (768–992)	2.5 (2.1–3.1)	4.1 (3.8–4.5)	6259 (5415–7235)	6331 (5474–7322)	63 (55–73)	377 (335–423)
	488–1431	1.5–5.0	3.0–6.2	3540–9979	3569–10,274	39–112	270–590
NAM	79 (49–127)	2.9 (2.4–3.4)	2.2 (2.0–2.5)	446 (259–769)	450 (262–773)	889 (517–1529)	2842 (1762–4584)
	18–324	1.5–5.0	1.7–3.4	69–2441	70–2449	163–5702	582–14,921
Mescaline 800 mg							
Mescaline	1721 (1482–1998)	2.2 (1.5–3.2)	3.7 (3.5–4.0)	13,047 (11,119–15,310)	13,144 (11,190–15,439)	61 (52–72)	328 (282–381)
	1021–2414	0.5–6.0	3.2–5.3	7274–17,485	7313–17,636	45–109	229–611
TMPAA	1532 (1294–1814)	3.0 (2.3–3.8)	4.2 (3.9–4.5)	11,554 (9435–14,149)	11,677 (9502–14,351)	69 (56–84)	413 (347–491)
	919–3007	1.0–6.0	3.4–6.2	6752–23,286	6784–23,502	34–118	236–666
NAM	159 (99–257)	3.4 (2.8–4.3)	2.2 (2.0–2.5)	980 (562–1707)	983 (565–1712)	814 (467–1416)	2621 (1637.7–4193)
	42–519	1.5–6.0	1.5–3.5	185–4529	186–4533	176–4292	649–10,779
Mescaline 800 mg + Ketanserin 40 mg							
Mescaline	2260 (1775–2879)	2.1 (1.6–2.7)	3.8 (3.6–4.0)	14,239 (11,654–17,396)	14,317 (11,721–17,489)	56 (46–68)	305 (247–377)
	771–3965	1.0–5.0	3.3–4.4	5212–20,987	5245–21,100	38–153	202–866
TMPAA	1564 (1253–1953)	2.9 (2.3–3.6)	4.1 (3.9–4.2)	11,910 (9492–14,944)	12,000 (9565–15,054)	67 (53–84)	390 (305–498)
	484–2935	1.0–6.0	3.5–4.6	4247–24,085	4285–24,327	33–187	182–1191
NAM	162 (93–282)	3.0 (2.5–3.5)	2.3 (2.1–2.5)	984 (525–1846)	988 (528–1851)	809 (432–1516)	2676 (1506–4753)
	31–809	1.5–5.0	1.7–3.5	175–6401	177–6404	125–4518	429–15,003

*AUC* area under the plasma concentration-time curve, *AUC*_*∞*_ AUC from time zero to infinity, *AUC*_*30*_ from time 0–30 h, *CL/F* apparent total clearance, *C*_*max*_ maximum observed plasma concentration, *total* after deglucuronidation (unconjugated + glucuronide); unconjugated, *t*_*1/2*_ plasma half-life, *t*_*max*_ time to reach Cmax, *95% CI* 95% confidence interval, *Vz/F* apparent volume of distribution, *TMPAA* 3,4,5-trimethoxyphenylacetic acid, *NAM* N-acetyl mescaline; data are geometric mean with 95% confidence interval of the mean and range; *n* = 16.

### Blinding

Results of the blinding assessment are shown in Supplementary Table S2. The 800 and 400 mg doses of mescaline were correctly identified by 16 and 15 participants, respectively, at the end-of-study visit. Two of the 16 subjects mistook 100 mg mescaline for placebo. All other doses of mescaline were identified as active doses. The mescaline and ketanserin combination was identified correctly by 12 subjects or mistaken as 100 mg mescaline (three subjects) or 200 mg mescaline (one subject) when they were asked after the sessions.

## Discussion

The present study was the first current investigation of acute effects of different doses of mescaline in healthy participants. We found that mescaline dose-dependently produced acute subjective responses without reaching a ceiling effect at the doses tested. Mescaline increased blood pressure, heart rate, body temperature, and pupil size and induced acute adverse effects, including headaches and nausea. Co-administration of the serotonin 5-HT_2A_ receptor antagonist ketanserin strongly reduced all acute effects of mescaline, indicating that acute psychedelic and physical effects of mescaline primarily depend on 5-HT_2A_ receptor activation. Additionally, we characterized pharmacokinetics of different doses of mescaline. Pharmacokinetics of mescaline were dose-proportional, with linear elimination kinetics and an elimination half-life of approximately 3.5 h. The relatively long duration of action of mescaline was attributable to slow absorption and consequently a long time to reach maximal effects.

A previous recent study characterized acute effects of 300 and 500 mg doses of mescaline in healthy subjects [14]. However, these two doses were administered in different participants using a parallel design. In contrast, the present study compared four different doses of mescaline, placebo, and the co-administration of mescaline and ketanserin within-subjects. We documented dose-dependent increases in subjective effects, including alterations of consciousness and mystical-type experiences starting at the 200 mg dose. Additionally, the effects were still increasing on practically all subjective effect measures when the dose was increased from 400 to 800 mg. Thus, no ceiling effect was observed at relatively high doses, in contrast to two recent studies with LSD [17, 35] and one study that used an identical design [17] and where 200 µg LSD did not produce more positive mood effects or more positive alterations of consciousness compared with 100 µg LSD. The present findings indicate that we were dosing within the linear range of the dose-effect curve with mescaline and that a ceiling effect for positive experiences may be reached only at higher doses. Confirming this view, a 500 mg dose of mescaline hydrochloride produced comparable effects to 100 µg LSD base or 20 mg psilocybin dihydrate when they were compared within the same study and subjects [14]; therefore, an 800 mg dose of mescaline could be expected to correspond to 160 µg LSD base or 32 mg psilocybin dihydrate. One might speculate that a higher 1000 mg dose of mescaline (corresponding to 200 µg LSD) might result in a ceiling effect. However, the 800 mg dose already produced substantial adverse effects, including relevant nausea and vomiting, and we expect tolerability of the 1000 mg mescaline dose to be low. In contrast, 100 mg of mescaline was chosen as the lowest dose administered in this study to elicit the threshold for inducing acute subjective effects. Dosages ranging from 178 to 356 mg of mescaline hydrochloride are considered moderate [36].

The present study also investigated the mechanism of action of mescaline in humans. Mescaline shows binding affinity for 5-HT_2A_ and 5-HT_1A_ and adrenergic α_2A_ receptors [2, 37]. Blocking only 5-HT_2A_ receptors with ketanserin reduced most acute effects of mescaline in the present study, indicating that 5-HT_2A_ receptors primarily mediated these responses. Typically, the subjects correctly identified the co-administration of mescaline and ketanserin because of the initial short-lasting effects of mescaline, followed by a marked and rapid decrease. The present study complements previous findings that ketanserin attenuated acute psychedelic effects of LSD and psilocybin in humans [17–19, 21]. Importantly, ketanserin was administered at the same time as mescaline in the present study and not before. In several participants, initial effects of mescaline appeared before sufficient ketanserin reached the circulation and could antagonize the mescaline response. Therefore, the remaining acute effects of mescaline, when co-administered with ketanserin, were partly attributable to the delayed kinetics and effect of ketanserin in some participants and not to a failure of ketanserin to dynamically block the mescaline response. However, minimal subjective effects of mescaline persisted after maximal ketanserin concentrations were reached, indicating a very minor contribution of other receptors to the mediation of acute effects of mescaline. The reason for using co-administration rather than the pre-administration of ketanserin in the present study was our concern that effects of mescaline may be reestablished later in the day because of the long duration of action of mescaline and historically reportedly much longer plasma half-life of mescaline (6 h) [38] compared with ketanserin (2 h for the clinically relevant early half-life) [39].

The present study was the first to use a range of different doses of mescaline to characterize its pharmacokinetics using modern analytics. Plasma concentrations (C_max_ and the area under the curve) increased dose-proportionally with increasing doses. The concentrations were slightly lower at the 400 and 800 mg doses of mescaline, very likely because of emesis at the mescaline effect onset, which may have removed some of the administered substance (4–8 capsules of 100 mg were administered to obtain these doses). The co-administration of ketanserin with mescaline reduced nausea and emesis and resulted in higher mescaline concentrations that were in the range that was expected based on dose-proportional extrapolation of the values of lower doses. We found that the order in which mescaline doses were administered had no significant effect on the subjective response with exception for nausea. This may be explained by the fact that subjects were sensitized for nausea after receiving a high dose first, which was more likely to induce nausea. However, it is unclear why mescaline induced more nausea compared with other classic serotonergic psychedelics having a similar receptor profile [40].

The present study found that mescaline produced moderate autonomic effects, including increases in blood pressure, heart rate, body temperature, and pupil size as previously reported [14] and very similar to LSD [14, 17] and psilocybin [14].

The present study confirmed the recently reported plasma elimination half-life of approximately 3.6 h [14], which is markedly shorter than the 6 h that was indicated by earlier studies [36, 38]. Consistent with the elimination half-life of mescaline of 3.6 h, doubling the dose of mescaline in the present study extended the effect duration by approximately 3 h. The average effect durations of mescaline were 10.7 and 14 h at the 400 and 800 mg doses, respectively. The average effect durations were 9.7 and 11.1 h at 300 and 500 mg mescaline, respectively, in a previous study [14]. By comparison, the effect durations of LSD were 8.3 and 11 h at 100 and 200 µg LSD, respectively, when measured similarly [17]. Notably, the longer effect duration of mescaline is not attributable to longer plasma elimination because the plasma elimination half-life of LSD is 4 h and comparable to mescaline [17]. The longer duration of action of mescaline can largely be explained by its longer time (2.2 h) to reach the peak plasma concentration compared with LSD (1.3–1.4 h) and the resulting longer time to reach peak responses for mescaline (3.2–4.0 h) compared with LSD (2.3–2.5 h) [14, 17].

The present study had several notable strengths. Four different doses of mescaline were administered within the same study and within-subjects and compared to a placebo in a controlled laboratory environment under double-blind conditions. Furthermore, a ketanserin-mescaline condition was included to shed light on the mechanism of action of mescaline in humans. We also improved blinding of the different conditions by using several active doses and the ketanserin condition. We included the same number of female and male participants. We administered internationally used, standardized, and validated psychometric assessments. Finally, plasma mescaline concentrations and pharmacokinetic parameters of pharmaceutically well-defined doses of mescaline were analyzed for all doses and up to 30 h. Ketanserin concentrations were also determined. Despite these strengths, the study also had limitations. We did not include doses higher than 800 mg mescaline. Ketanserin was administered at the same time as mescaline and not as a pretreatment. All but one participant had prior experience with psychedelics, although no one had used them more than 15 times. Finally, the study was conducted in a highly regulated environment and involved only healthy people, meaning that responses to mescaline may differ among individuals in other settings and those with psychiatric conditions.

## Conclusion

The present study characterized acute subjective and autonomic effects of different doses of mescaline. Starting at the 200 mg dose of mescaline, it produced dose-dependent psychedelic effects up to 800 mg, with no ceiling effect. The 800 mg dose of mescaline produced significant subjective bad drug effects and emesis. The present mescaline dose-response data may be useful for defining doses in future psychedelic research in healthy subjects and patients. The co-administration of ketanserin together with 800 mg mescaline attenuated the acute response, confirming that serotonin 5-HT_2A_ receptors are primarily involved in psychedelic effects of mescaline in humans.

## Supplementary information

Supplement

## Data Availability

The datasets presented in this article are not readily available because the data associated with this work are owned by the University Hospital Basel and were licensed by Mind Medicine. Requests to access the datasets should be directed to MEL, matthias.liechti@usb.ch.
