# Supplementary material for: Acute dose-dependent effects of mescaline in a double-blind placebo-controlled study in healthy subjects
Source: Transl Psychiatry. 2024 Sep 30;14:395. doi: 10.1038/s41398-024-03116-2 (PMC11442856; doi:10.1038/s41398-024-03116-2)
Supplement: Supplementary file 1 — Supplement [file 41398_2024_3116_MOESM1_ESM.docx]

**Supplement**

**Methods**

*Participants*

*Use of medications during study inclusion*

The use of medications that may interfere with the study medications (e.g., antidepressants, antipsychotics, or sedatives) was not allowed. Medications that were not expected to interfere with the study drug were allowed. Eleven participants have used the following medications during study inclusion: Analgesics (including ibuprofen, paracetamol, carbasalate calcium, etofenamate and naproxen), antitussive (dextromethorphan), proton pump inhibitors (esomeprazole), corticosteroids (budesonide), antidiarrheals (saccharomyces boulardii), monoclonal antibody treatment (ustekinumab), and antibiotics (fosfomycin). None of these drugs were taken on actual study days.

*Subjective drug effects measurements*

*Visual Analog Scales (VASs)*

Subjective effects were assessed repeatedly using visual analog scales (VASs) [1,2] 1 h before and 0, 0.5, 1, 1.5, 2, 3, 4, 5, 6, 8, 9, 10, 11, 12, 14, 16, 20, 24 and 30 h after drug administration. The VASs included “any drug effect”, “good drug effect”, “bad drug effect”, “stimulated”, “happy”, “fear”, “nausea”, “visual alteration”, “auditory alterations”, “sounds seem to influence what I see” (= audio-visual synesthesia), “altered perception of time”, “the boundaries between myself and my surroundings seem to blur” (= ego dissolution), “I am having insights into connections that previously puzzled me” (= insights), “open”, “talkative”, and “trust” [1,3]. The VASs were presented as 100-mm horizontal lines (0 - 100%), marked from “not at all” on the left to “extremely” on the right. The VAS “open”, “talkative”, and “trust” were bidirectional and marked with “normal” in the middle at 50 mm and “not at all” on the left (0 mm) and “extremely” (100 mm) on the right. The primary VAS outcome measures were “any drug effect”, “good drug effect”, and “bad drug effect”. These VASs have been repeatedly used and shown to be with other psychedelics like lysergic acid diethylamide (LSD) or psilocybin [1,2,4,5]. The VASs could be completed easily and rapidly by the participants under the influence of mescaline allowing to define the drug effect over time. The VAS items are simple instruments. The VAS “any drug effect” is an overall effect measure to characterize the overall effect intensity and time course. The VAS “good drug effect” is an overall measure of effects subjectively considered positive and interrelated with other measures such as “drug liking”. The VAS “bad drug effect” is an overall measure of subjectively considered negative effects.

*5 Dimensions of Altered States of Consciousness (5D-ASC) scale*

The 5 Dimensions of Altered States of Consciousness (5D-ASC) scale [6,7] was used as one primary outcome measure and was administered 30 h after drug administration to retrospectively rate the psychedelic experience. The 5D-ASC contains 94 items represented by visual analog scales. The 94 items may be clustered into five subscales/dimensions [6] and 11 lower-order subscales [7]. The 5D-ASC dimension “oceanic boundlessness” (27 items) measures derealization and depersonalization associated with positive emotional states, ranging from heightened mood to euphoric exaltation. The corresponding lower-order subscales include “experience of unity,” “spiritual experience,” “blissful state,” “insightfulness,” and “disembodiment”. The dimension “anxious ego dissolution” (21 items) summarizes ego-disintegration and loss of self-control phenomena associated with anxiety. The corresponding lower-order scales include “impaired control of cognition” and “anxiety.” The dimension “visionary restructuralization” (18 items) consists of the lower-order scales “complex imagery,” “elementary imagery,” “audio-visual synesthesia,” and “changed meaning of percepts”. Two additional dimensions describe “auditory alterations” (15 items) and “reduction of vigilance” (12 items). The total 3D-ASC score is calculated by adding the scores of the three main dimensions “oceanic boundlessness”, “anxious ego-dissolution”, and “visionary restructuralization” and can be interpreted as a measure for the general intensity of alteration of the mind [8]. The scale is well-validated in German [6] and many other languages and is widely used to characterize the subjective effects of various psychedelic substances. In particular, the scale has been used by most research groups investigating psychedelics [1,2,9-13]. Ratings on the 5D-ASC have been shown to closely correlate with ratings on the Mystical Effects Questionnaire (MEQ, see below) [8].

*Mystical Effects Questionnaire (MEQ30)*

Mystical experiences were assessed 30 h after drug administration using the 100-item States of Consciousness Questionnaire (SOCQ) [8,14]. The SOCQ includes the 43-item Mystical Effects Questionnaire (MEQ43) [14], 30-item Mystical Effects Questionnaire (MEQ30) [15], and subscales for “aesthetic experience” and negative “nadir” effects. The MEQ has been used in numerous clinical trials with psychedelics [8,14,16-26]. The items of both the MEQ30 and the MEQ43 provide scale scores for seven domains of mystical experiences: Internal unity, external unity, sacredness, noetic quality (as real as or “more real” than everyday reality), deeply felt positive mood, transcendence of time and space, and ineffability/paradoxicality (difficulty describing the experience in words). The sum of all scale scores was used as an overall measure of the mystical-type experience. We also derived the four scale scores of the revised 30-item MEQ: Mystical experience, positive mood, transcendence of time and space, and ineffability [15]. A complete mystical experience was defined as scores ≥ 60% on all MEQ30 factors [15]. For the scale validation, see [15]. For an analysis of the interrelation of the MEQ and the 5D-ASC in regard to responses to LSD, see [8]. For the German translation of the MEQ30, see the online supplement of [8].

*Adjective Mood Rating Scale (AMRS)*

The Adjective Mood Rating Scale (AMRS) [27] was used 1 h before and 4, 8, 14, and 30 h after drug administration. The AMRS is a validated 60-item Likert rating scale, used primarily in Europe and consists of subscales including ratings on “well-being”, “anxiety”, “inactivity”, “extraversion”, “introversion”, and “emotional excitation”. It is suitable for repeated measurements of mood states. The short German EWL60S version was used [27]. The completion of the ratings under the effects of psychedelic substances is possible but difficult because it lasts several minutes. The scale was mainly used in paper and pencil version and sometimes completed verbally during states of markedly impaired concentration during strong psychoactive effects. The AMRS was included as a secondary supportive measure because it could be considered a better validated measure of mood states and producing more defined ratings than the VASs and to support findings on the VASs (AMRS well-being considered similar to VAS “good drug effect”; AMRS anxiety considered similar to VAS “fear”).

*Study drugs*

Mescaline hydrochloride (99.3% purity; ReseaChem GmbH, Burgdorf, Switzerland) was administered in opaque capsules produced according to Good Manufacturing Practice in units containing 100 mg mescaline. The exact analytically confirmed mescaline content (mean ± SD) was 94.96 ± 0.08 mg (*n* = 6 samples). Ketanserin was obtained as the marketed drug Ketensin (20 mg, Janssen-Cilag, Leiden, NL) and encapsulated with opaque capsules to ensure blinding. Each capsule contained 20 mg of ketanserin tartrate. Placebo consisted of identical opaque capsules that were filled with mannitol. A double-dummy method was used. All subjects received ten capsules in each session: (*i*) ten placebo capsules, (*ii*) nine placebo capsules and 100 mg mescaline, (*iii*) eight placebo capsules and two 100 mg mescaline capsules, (*iv*) six placebo capsules and four 100 mg mescaline capsules, (*v*) two placebo capsules and eight 100 mg mescaline capsules, and (*vi*) two ketanserin capsules and eight 100 mg mescaline capsules.

**Results**

**
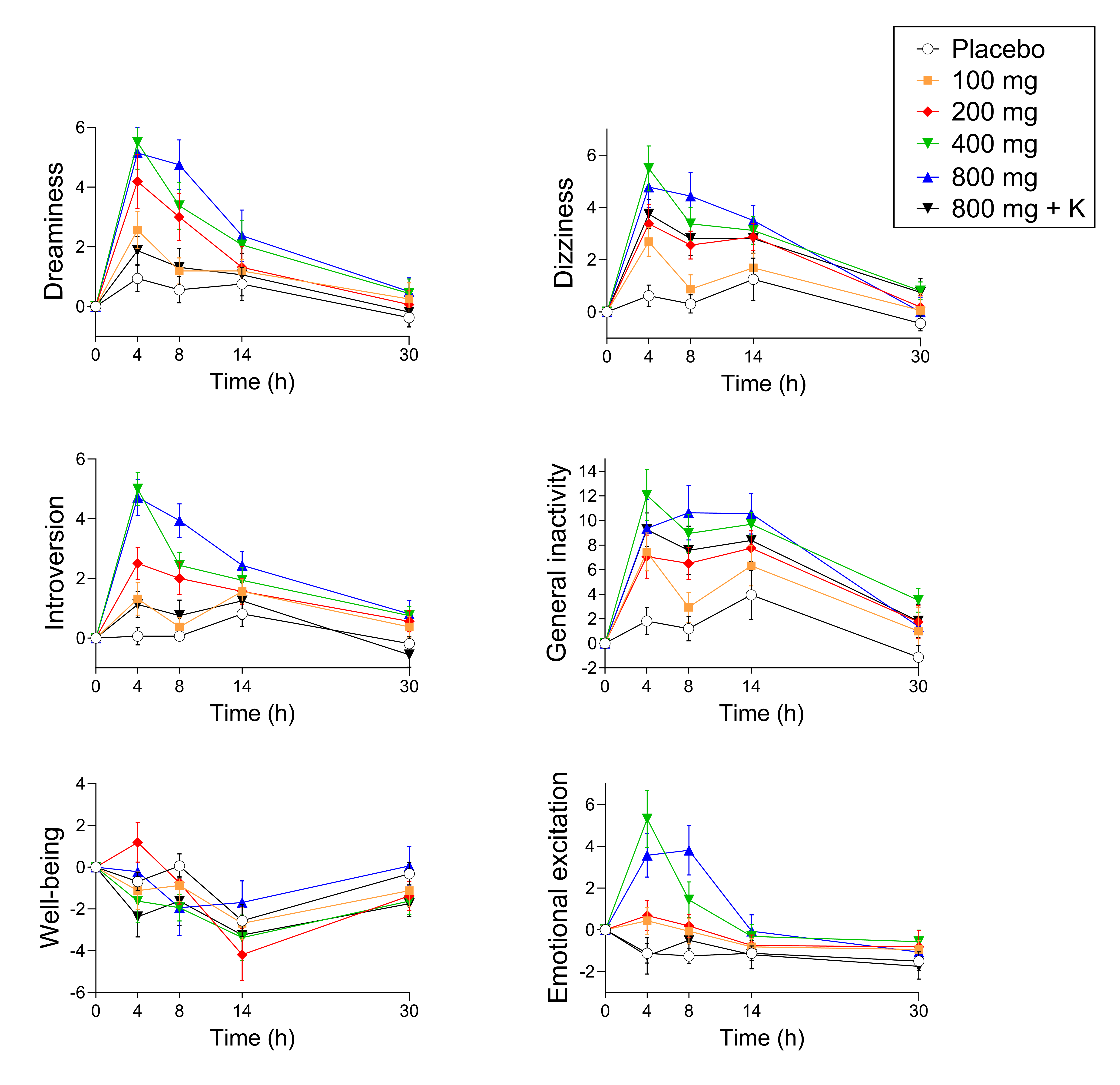
**

**Figure S1.** Subjective effects over time on the Adjective Mood Rating Scale (AMRS). Mescaline increased ratings of dreaminess, dizziness, introversion, general inactivity, and emotional excitation compared with placebo. Ketanserin (K) co-administration reduced dreaminess, introversion, and emotional excitation compared with mescaline 800 mg alone. Mescaline and ketanserin were administered at t = 0 h. The data are expressed as mean ± SEM changes from baseline. The corresponding maximal effects and statistics are shown in Supplementary Table S1.


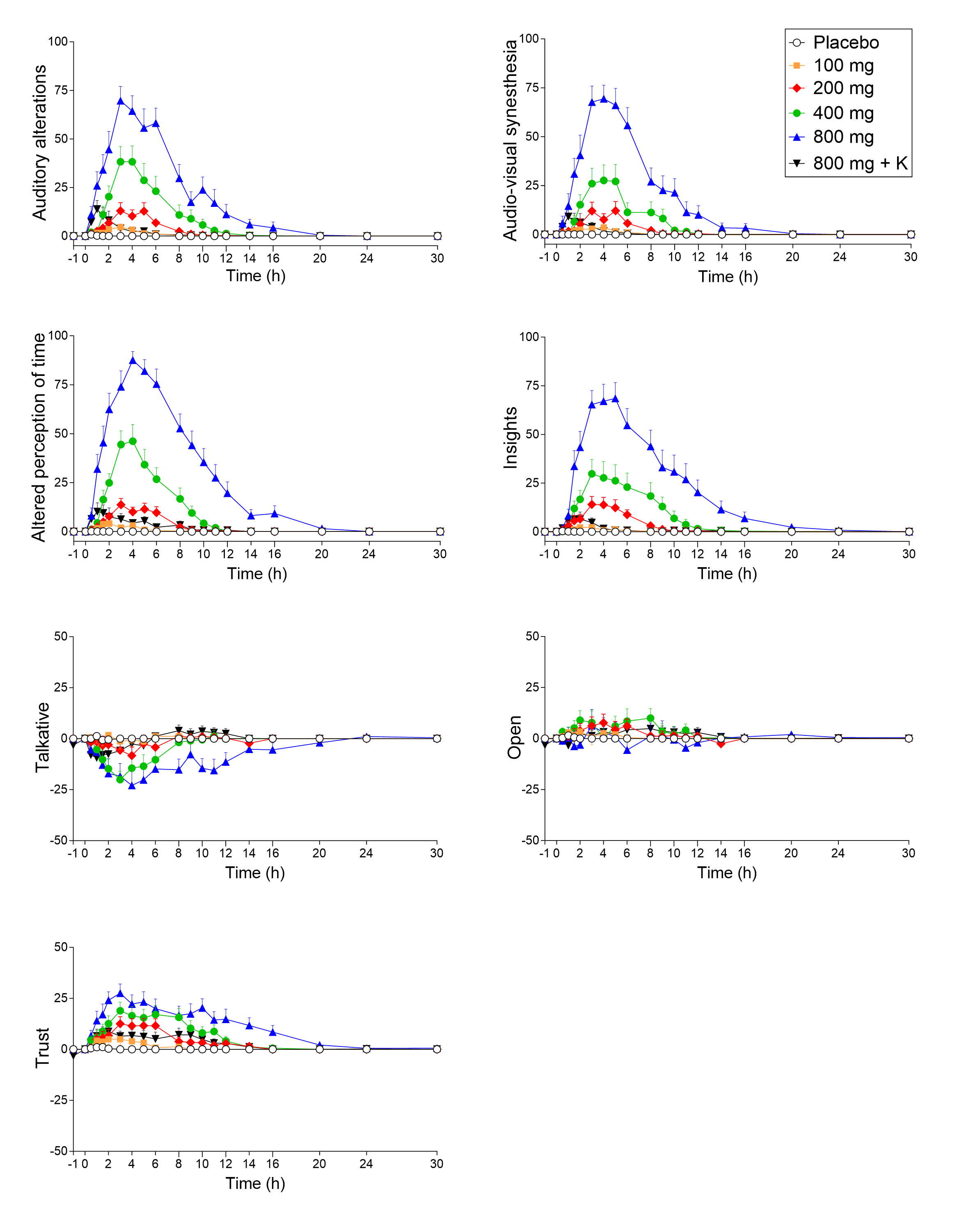


**Figure S2.** Acute subjective effects of mescaline on the VASs. Mescaline dose-dependently increased ratings of “auditory alterations”, “audio-visual synesthesia”, “altered perception of time”, “insights”, “open” and “trust” and decreased ratings of “talkative” compared with placebo. Ketanserin (K) reduced the effects of mescaline on the VASs. Mescaline and ketanserin were administered at t = 0 h. The data are expressed as mean ± SEM changes from baseline. Maximal effects and statistics are shown in Supplementary Table S1.


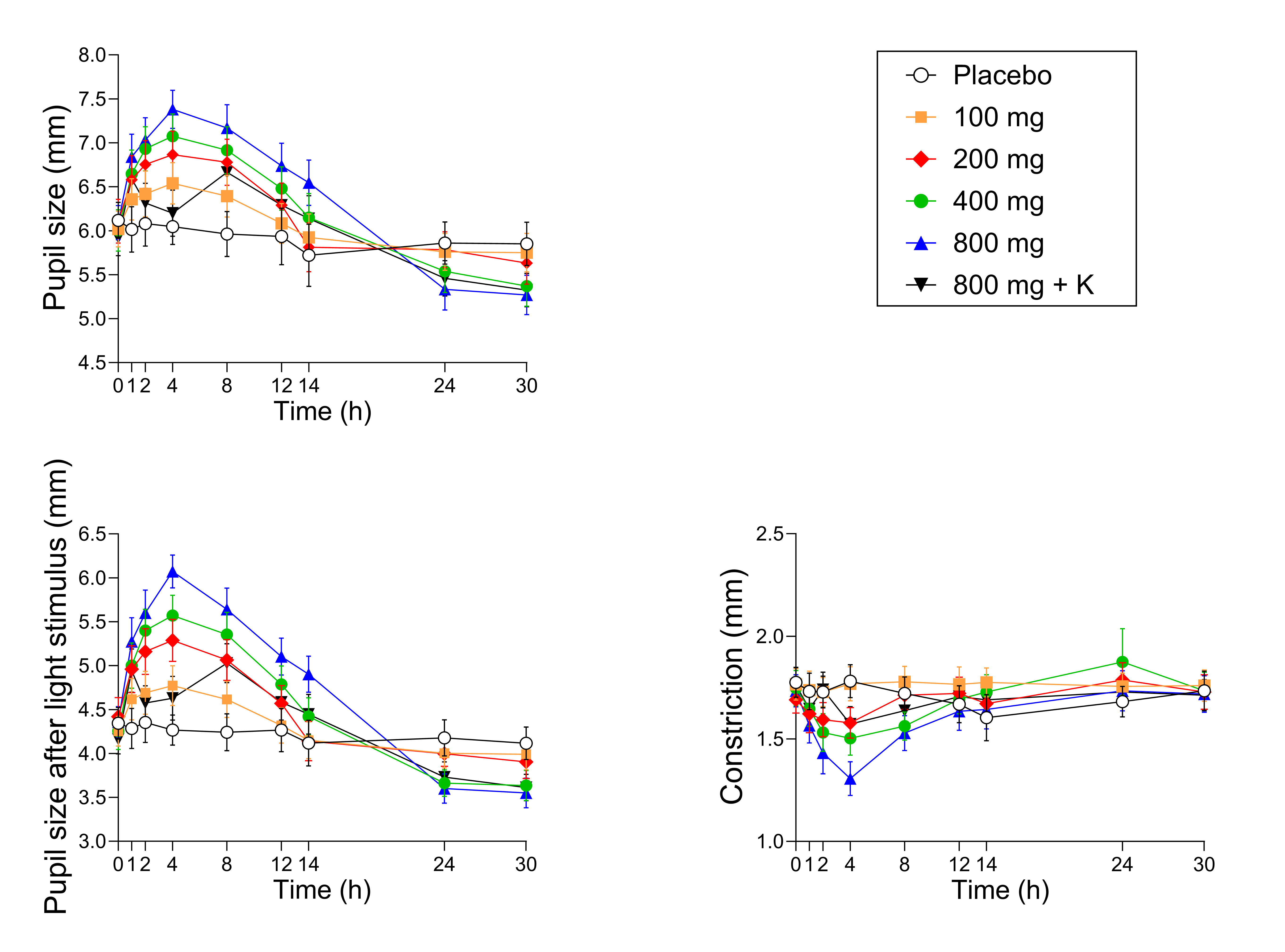


**Figure S3.** Effects of mescaline on pupillary function. Mescaline increased pupil size dose-dependently at rest and after a light stimulus and reduced the constriction amplitude. Ketanserin (K) reversed the mescaline-induced alterations of pupillary function. Mescaline and ketanserin were administered at t = 0 h. The data are expressed as mean ± SEM changes from baseline. Maximal effects and statistics are shown in Supplementary Table S1.

**
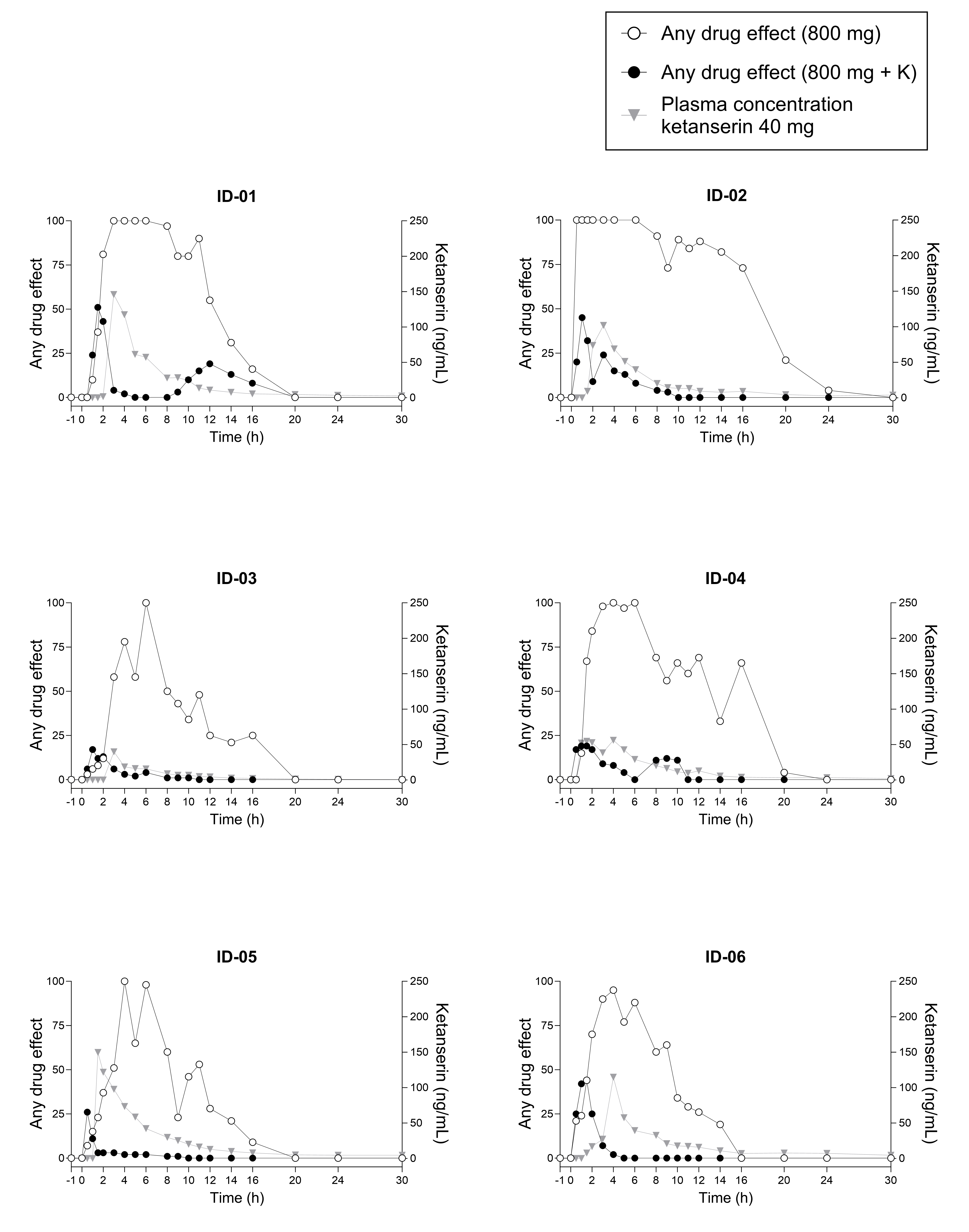
**

**
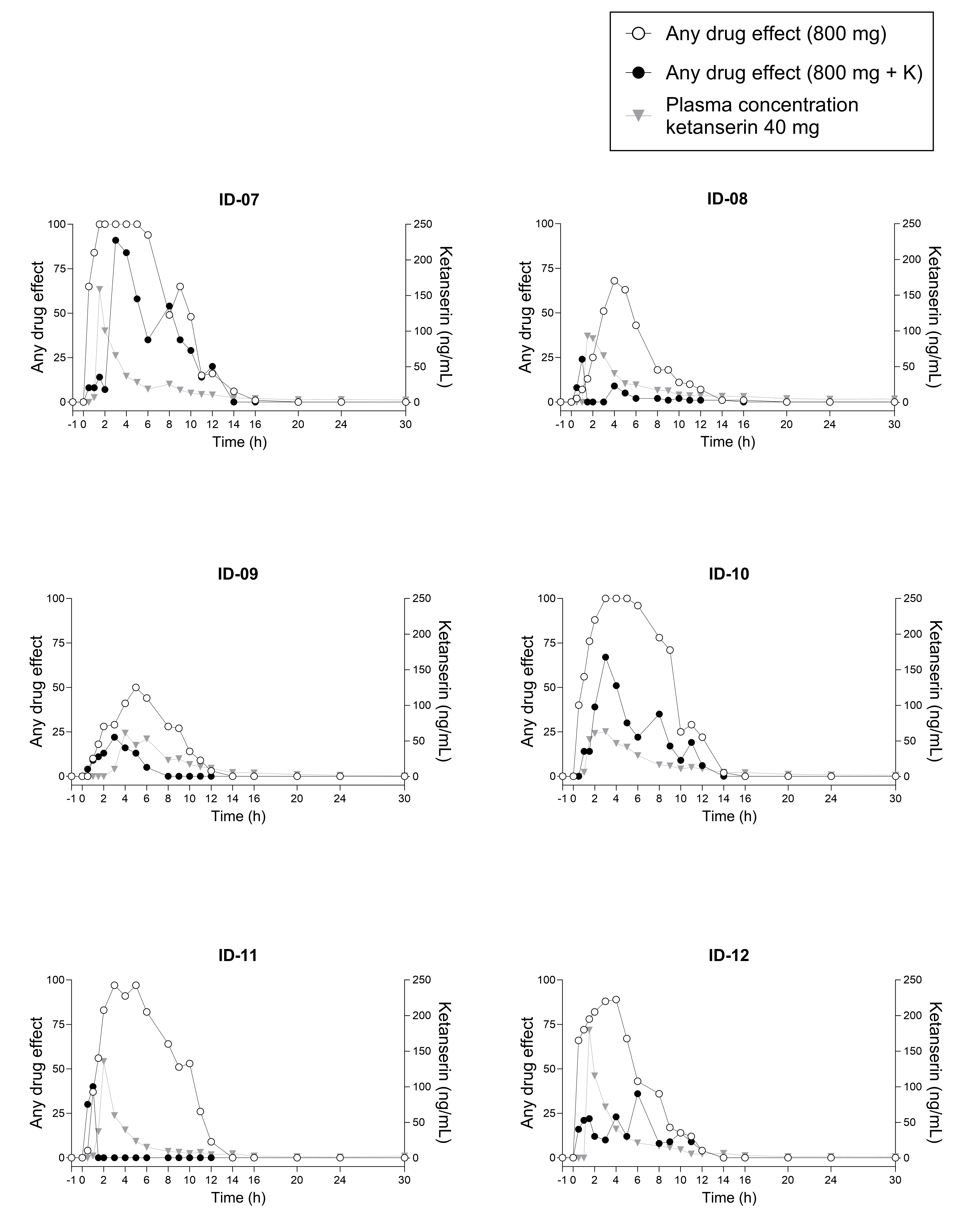
**

**
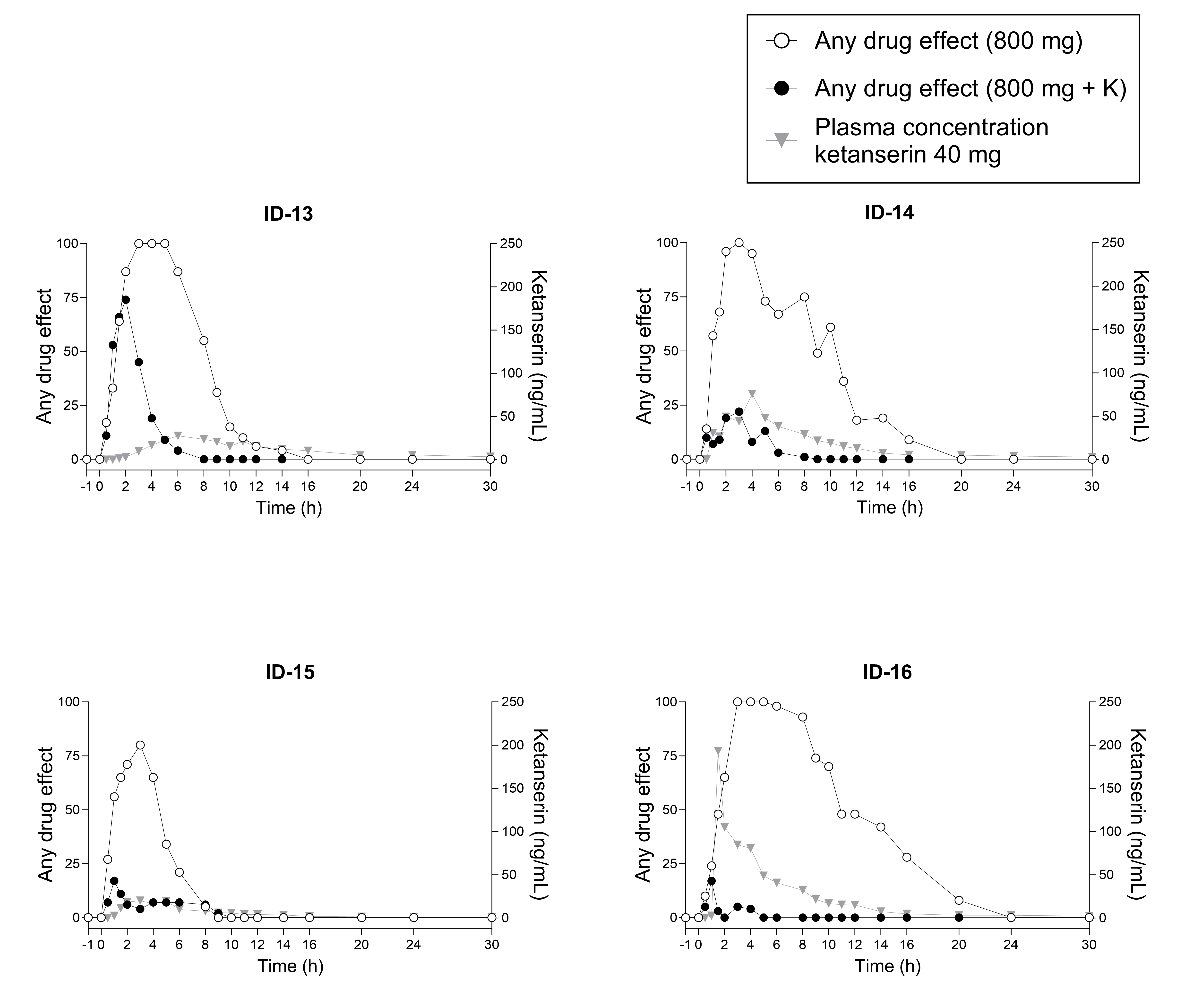
**

**Figure S4.** Individual subjective effect ratings (any drug effect) of mescaline (800 mg) over time with and without ketanserin (K) in relation to the plasma concentration of ketanserin. Ketanserin reduced the subjective effects of mescaline. In several participants, mescaline effects started to develop up to 2 h and were then reversed by ketanserin once its plasma concentrations increased. Overall, ketanserin blocked the acute effects of mescaline completely in 11 subjects and partly in 5 subjects once it was present in the plasma. Mescaline and ketanserin were administered at the same time at t = 0 h. Data are individual values shown for each subject in separate plots.

**References**

1 Holze F, Vizeli P, Muller F, Ley L, Duerig R, Varghese N, et al. Distinct acute effects of LSD, MDMA, and D-amphetamine in healthy subjects. Neuropsychopharmacology. 2020;45(3):462-71.

2 Schmid Y, Enzler F, Gasser P, Grouzmann E, Preller KH, Vollenweider FX, et al. Acute effects of lysergic acid diethylamide in healthy subjects. Biol Psychiatry. 2015;78(8):544-53.

3 Hysek CM, Schmid Y, Simmler LD, Domes G, Heinrichs M, Eisenegger C, et al. MDMA enhances emotional empathy and prosocial behavior. Social cognitive and affective neuroscience. 2014;9:1645-52.

4 Holze F, Duthaler U, Vizeli P, Muller F, Borgwardt S, Liechti ME. Pharmacokinetics and subjective effects of a novel oral LSD formulation in healthy subjects. British journal of clinical pharmacology. 2019;85:1474-83.

5 Dolder PC, Schmid Y, Steuer AE, Kraemer T, Rentsch KM, Hammann F, et al. Pharmacokinetics and pharmacodynamics of lysergic acid diethylamide in healthy subjects. Clin Pharmacokinetics. 2017;56:1219-30.

6 Dittrich A. The standardized psychometric assessment of altered states of consciousness (ASCs) in humans. Pharmacopsychiatry. 1998;31 (Suppl 2):80-4.

7 Studerus E, Gamma A, Vollenweider FX. Psychometric evaluation of the altered states of consciousness rating scale (OAV). PLoS One. 2010;5(8):e12412.

8 Liechti ME, Dolder PC, Schmid Y. Alterations in conciousness and mystical-type experiences after acute LSD in humans. Psychopharmacology. 2017;234:1499-510.

9 Carhart-Harris RL, Kaelen M, Bolstridge M, Williams TM, Williams LT, Underwood R, et al. The paradoxical psychological effects of lysergic acid diethylamide (LSD). Psychological medicine. 2016;46:1379-90.

10 Dolder PC, Schmid Y, Mueller F, Borgwardt S, Liechti ME. LSD acutely impairs fear recognition and enhances emotional empathy and sociality. Neuropsychopharmacology. 2016;41:2638-46.

11 Bershad AK, Schepers ST, Bremmer MP, Lee R, de Wit H. Acute subjective and behavioral effects of microdoses of lysergic acid diethylamide in healthy human volunteers. Biol Psychiatry. 2019;86(10):792-800.

12 Preller KH, Herdener M, Pokorny T, Planzer A, Kraehenmann R, Stämpfli P, et al. The fabric of meaning and subjective effects in LSD-induced states depend on serotonin 2A receptor activation Curr Biol. 2017;27:451-57.

13 de Deus Pontual AA, Senhorini HG, Corradi-Webster CM, Tofoli LF, Daldegan-Bueno D. Systematic review of psychometric instruments used in research with psychedelics. J Psychoactive Drugs. 2022:1-10.

14 Griffiths RR, Richards WA, McCann U, Jesse R. Psilocybin can occasion mystical-type experiences having substantial and sustained personal meaning and spiritual significance. Psychopharmacology (Berl). 2006;187(3):268-83; discussion 84-92.

15 Barrett FS, Johnson MW, Griffiths RR. Validation of the revised Mystical Experience Questionnaire in experimental sessions with psilocybin. J Psychopharmacol. 2015;29(11):1182-90.

16 MacLean KA, Johnson MW, Griffiths RR. Mystical experiences occasioned by the hallucinogen psilocybin lead to increases in the personality domain of openness. J Psychopharmacol. 2011;25(11):1453-61.

17 Griffiths RR, Johnson MW, Richards WA, Richards BD, McCann U, Jesse R. Psilocybin occasioned mystical-type experiences: immediate and persisting dose-related effects. Psychopharmacology. 2011;218(4):649-65.

18 Griffiths R, Richards W, Johnson M, McCann U, Jesse R. Mystical-type experiences occasioned by psilocybin mediate the attribution of personal meaning and spiritual significance 14 months later. J Psychopharmacol. 2008;22(6):621-32.

19 Garcia-Romeu A, Griffiths RR, Johnson MW. Psilocybin-occasioned mystical experiences in the treatment of tobacco addiction. Current drug abuse reviews. 2014;7(3):157-64.

20 Garcia-Romeu A, Davis AK, Erowid F, Erowid E, Griffiths RR, Johnson MW. Cessation and reduction in alcohol consumption and misuse after psychedelic use. J Psychopharmacol. 2019;33(9):1088-101.

21 Griffiths RR, Johnson MW, Richards WA, Richards BD, Jesse R, MacLean KA, et al. Psilocybin-occasioned mystical-type experience in combination with meditation and other spiritual practices produces enduring positive changes in psychological functioning and in trait measures of prosocial attitudes and behaviors. J Psychopharmacol. 2018;32:49-69.

22 Griffiths RR, Johnson MW, Carducci MA, Umbricht A, Richards WA, Richards BD, et al. Psilocybin produces substantial and sustained decreases in depression and anxiety in patients with life-threatening cancer: a randomized double-blind trial. J Psychopharmacol. 2016;30(12):1181-97.

23 Ross S, Bossis A, Guss J, Agin-Liebes G, Malone T, Cohen B, et al. Rapid and sustained symptom reduction following psilocybin treatment for anxiety and depression in patients with life-threatening cancer: a randomized controlled trial. J Psychopharmacol. 2016;30(12):1165-80.

24 Ley L, Holze F, Arikci D, Becker AM, Straumann I, Klaiber A, et al. Comparative acute effects of mescaline, lysergic acid diethylamide, and psilocybin in a randomized, double-blind, placebo-controlled cross-over study in healthy participants. Neuropsychopharmacology. 2023.

25 Holze F, Vizeli P, Ley L, Muller F, Dolder P, Stocker M, et al. Acute dose-dependent effects of lysergic acid diethylamide in a double-blind placebo-controlled study in healthy subjects. Neuropsychopharmacology. 2021;46(3):537-44.

26 Vogt SB, Ley L, Erne L, Straumann I, Becker AM, Klaiber A, et al. Acute effects of intravenous DMT in a randomized placebo-controlled study in healthy participants. Transl Psychiatry. 2023;13(1):172.

27 Janke W, Debus G. Die Eigenschaftswörterliste. Hogrefe: Göttingen.; 1978.
